# Serum Endocan Levels Correlate with Metabolic Syndrome Severity and Endothelial Dysfunction: A Cross-Sectional Study Using the MetS-Z Score

**DOI:** 10.3390/metabo15080521

**Published:** 2025-08-01

**Authors:** Mehmet Vatansever, Selçuk Yaman, Ahmet Cimbek, Yılmaz Sezgin, Serap Ozer Yaman

**Affiliations:** 1 Department of Family Medicine, Kanuni Training and Research Hospital, University of Health Sciences, Trabzon 61080, Türkiye; 2 Department of Medical Biochemistry, Trabzon Kanuni Training and Research Hospital, University of Health Sciences, Trabzon 61080, Türkiye; 3 Department of Internal Medicine, Kanuni Training and Research Hospital, University of Health Sciences, Trabzon 61080, Türkiye

**Keywords:** endocan, endothelial cells, metabolic syndrome, inflammation, risk stratification

## Abstract

**Background:** Metabolic syndrome (MetS) is a complex clinical condition characterized by the coexistence of interrelated metabolic abnormalities that significantly increase the risk of cardiovascular diseases and type 2 diabetes mellitus. Endocan—an endothelial cell-specific molecule—is considered a biomarker of endothelial dysfunction and inflammation. This study aimed to evaluate the relationship between serum endocan levels and the severity of MetS, assessed using the MetS-Z score. **Methods:** This study included 120 patients with MetS and 50 healthy controls. MetS was diagnosed according to the NCEP-ATP III criteria. MetS-Z scores were calculated using the MetS Severity Calculator. Serum levels of endocan, sICAM-1, and sVCAM-1 were measured using the ELISA method. **Results:** Serum levels of endocan, sICAM-1, and sVCAM-1 were significantly higher in the MetS group compared to the control group (all *p* < 0.001). When the MetS group was divided into tertiles based on MetS-Z scores, stepwise and statistically significant increases were observed in the levels of endocan and other endothelial markers from the lowest to highest tertile (*p* < 0.0001). Correlation analysis revealed a strong positive association between the MetS-Z score and serum endocan levels (r = 0.584, *p* < 0.0001). ROC curve analysis showed that endocan has high diagnostic accuracy for identifying MetS (AUC = 0.967, *p* = 0.0001), with a cutoff value of >88.0 ng/L. **Conclusions:** Circulating levels of endocan were significantly increased in MetS and were associated with the severity of MetS, suggesting that endocan may play a role in the cellular response to endothelial dysfunction-related injury in patients with MetS.

## 1. Introduction

Metabolic syndrome (MetS) is a cluster of interrelated metabolic abnormalities—including abdominal obesity, hypertension, insulin resistance, dyslipidemia, and glucose intolerance—that collectively heighten the risk for type 2 diabetes mellitus (T2DM) and atherosclerotic cardiovascular disease (ASCVD) [1,2]. The increasing global prevalence of MetS—affecting over one-third of adults in many populations—represents a major public health concern [3]. Among the many mechanisms linking MetS to adverse cardiovascular outcomes, endothelial dysfunction has emerged as a central pathophysiological component [4,5]. This dysfunction reflects a pro-inflammatory, pro-thrombotic, and vasoconstrictive shift in endothelial phenotype, driven by oxidative stress, insulin resistance, and chronic low-grade inflammation [6]. As a result, there is an increasing need for early and reliable biomarkers that reflect vascular health in MetS patients and may support risk stratification and early intervention. Researchers have encountered challenges in delivering precise risk assessments for individuals with MetS, prompting the creation of a new MetS severity Z-score (MetS-Z) for additional research. The MetS-Z was derived from validated equations established through clinical trials, utilizing body mass index (BMI), high-density lipoprotein cholesterol (HDL), systolic blood pressure (SBP), triglycerides (TG), and fasting blood glucose (FBG). Studies have indicated that the MetS-Z score may correlate with diabetes and coronary heart disease, thereby offering supplementary predictive ability for these conditions [3]. Endothelial dysfunction, platelet hyperactivity, oxidative stress, and low-grade inflammation are some of the factors associated with the negative effects of this disorder on the vascular wall. Increased vasoconstriction and atherosclerosis occur as a result of the activation of these processes, eventually encouraging a prothrombotic condition [4]. According to clinical research, the development of atherosclerotic vascular disorders is significantly influenced by endothelial dysfunction, hyperlipidemia, oxidative stress, and platelet hyperactivity (2). All the factors that contribute to MetS negatively impact the endothelium. Endothelial dysfunction may raise the risk of insulin resistance and type 2 diabetes, in addition to contributing to the pathophysiology of atherosclerosis [4,5].

Dyslipidemia (high blood triglyceride and cholesterol levels) is an important risk factor for the development of atherosclerotic cardiovascular disease (CVD) [6]. It has been suggested that the presence of increased and prolonged atherogenic chylomicron remnants, decreased HDL levels, and the activation of leukocytes and endothelial cells due to the effects of these remnants are effective in altering the predisposition of individuals with MetS to atherosclerosis [6,7]. Increased dyslipidemia in MetS has been demonstrated to activate leukocytes, thus increasing oxidative stress and levels of pro-inflammatory cytokines which, in turn, lead to endothelial dysfunction [7]. Vascular endothelial cells play fundamental roles in processes such as inflammation, hemostasis, angiogenesis, and tumor invasion, due to their variety of secreted mediators and receptor/ligand interactions [8]. Endocan—also known as endothelial cell-specific molecule-1 (ESM-1)—is a dermatan sulfate proteoglycan secreted by activated vascular endothelial cells. It is known to participate in inflammation, endothelial activation, and neovascularization [9,10]. Elevated circulating endocan levels have been described in various inflammatory and cardiometabolic disorders, including obesity, T2DM, chronic kidney disease, and coronary artery disease [11,12,13]. Recent studies in 2024 have linked endocan to increased oxidative stress and integrin-mediated leukocyte adhesion, suggesting that it may play both a marker and a mediator role in endothelial dysfunction [14,15].

Understanding the relationship between endothelial dysfunction markers such as endocan and metabolic syndrome through metabolic syndrome severity scores may provide valuable contributions to combat this syndrome, which is considered an important independent risk factor for cardiovascular events. While several studies have independently established associations between endocan and individual metabolic or vascular risk factors, few investigations have systematically assessed the relationship between endocan and comprehensive MetS severity scores. In particular, the recently developed Metabolic Syndrome Severity Z-score (MetS-Z) provides a continuous, sex- and ethnicity-specific index of MetS burden that better captures subclinical disease and cardiometabolic risk than binary MetS criteria [16,17,18]. To date, however, the link between MetS-Z score and serum endocan levels has not been explored. Therefore, the aim of the present study was to examine the association between serum endocan levels and MetS severity as quantified by the MetS-Z score. We also aimed to determine whether elevated endocan levels correlate with other markers of endothelial dysfunction (sICAM-1, sVCAM-1) and assess their diagnostic utility. By stratifying patients according to MetS severity tertiles, we sought to evaluate the potential value of endocan as a marker of progressive endothelial activation in MetS.

## 2. Materials and Methods

### 2.1. Study Population

This cross-sectional study comprised 120 participants with metabolic syndrome and 50 healthy controls who had been admitted to our Family Medicine and Internal Medicine outpatient clinics. This study was conducted in accordance with the Declaration of Helsinki principles and received approval from the ethics review committee of the University of Health Sciences Trabzon Medicine Faculty (Ethical Committee for Scientific Research on Humans; Reference number: 2024/93; Approval Date: 23 July 2024). Informed written consent was acquired from all patients before their enrollment in the trial.

The exclusion criteria included the presence of cardiac and systemic conditions, including ischemic heart disease, congenital heart defects, valvular heart disease, neoplastic disorders, inflammatory diseases, and infectious diseases.

Participants taking medications potentially affecting endocan or endothelial function were excluded that could potentially interfere with the measurement of endocan and endothelial dysfunction, including treatments known to affect lipid and lipoprotein levels (e.g., angiotensin-converting enzyme inhibitors and statins), oral antidiabetic medications, or insulin; if they had a serious medical condition such as chronic kidney disease or hepatic failure; a history of gastric or intestinal surgery or inflammatory bowel disease; or if they were alcohol abusers, smokers, using any dietary supplements, or were pregnant or lactating.

**Figure 1:**
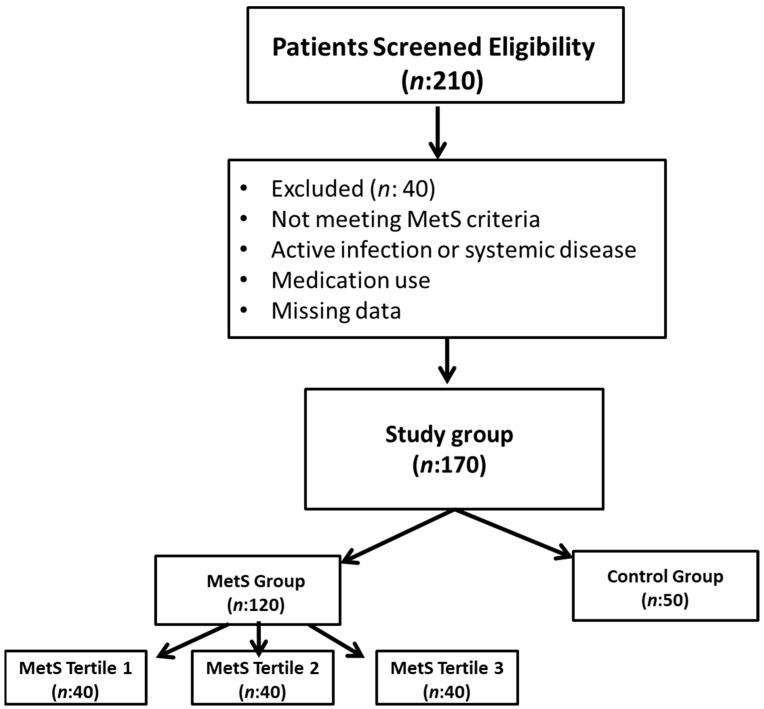
Participant flow chart.

The diagnostic criteria for metabolic syndrome were those endorsed by the National Cholesterol Education Program. The Expert Panel on Detection, Evaluation, and Treatment of High Blood Cholesterol in Adults—Adult Treatment Panel III (NCEP-ATP III) judgment is characterized by the presence of a minimum of three of the following components: (1) Augmented waist circumference (>102 cm for males, >88 cm for females); (2) elevated triglycerides (≥150 mg/dL) or utilization of triglyceride-lowering medications; (3) diminished HDL cholesterol (<40 mg/dL in males, <50 mg/dL in females); (4) hypertension (≥130/≥85 mm Hg) or administration of antihypertensive medications; and (5) fasting glucose (≥100 mg/dL) or use of antidiabetic medications [16]. Figure 1 shows the flowchart of participants through the study.

### 2.2. Assessment of MetS Severity Z-Score (MetS-Z Score)

The Metabolic Syndrome Severity Score (MetS-S), derived from BMI, was used to assess the risk of MetS in the present study. The MetS-S calculation was conducted utilizing the online resource MetS Severity Calculator (available at https://metscalc.org/) (accessed on 15 January 2025). The MetS-S calculator is an online tool for computation of an individual’s MetS-S score utilizing proven and thoroughly researched equations. MetS Calc was created by Dr. Matthew J. Gurka from the University of Florida and Dr. Mark DeBoer from the University of Virginia under CTS-IT. The formulas for calculating MetS-S were derived from the National Health and Nutrition Examination Survey (NHANES) study in the USA, incorporating factors such as age, race, gender, BMI, triglycerides, high-density lipoprotein cholesterol (HDL-C), systolic blood pressure (SBP), and blood glucose levels. The MetS-Severity Score was computed in two forms, including MetS-S zero (MetS-Sz), which spans from negative infinity to positive infinity [17,18].

### 2.3. Physical and Laboratory Assessments

Anthropometric measurements (weight, height, waist circumference) and systemic blood pressure readings were obtained via physical examination in accordance with standard protocols. The body mass index (BMI) was calculated by calculating weight (kg) divided by height squared (m^2^) [19]. Waist circumference was measured in the horizontal plane at the point halfway between the smallest rib and the iliac crest [20]. Resting systolic and diastolic blood pressure values were measured three times at one-minute intervals using a standard mercury sphygmomanometer after a five-minute rest period. The average of the second and third readings was employed in the analysis. Venous blood samples were collected in the morning after a minimum fasting period of 8 h. Serum cholesterol, triglycerides, and high-density lipoprotein cholesterol (HDL-C) were measured using enzymatic colorimetric techniques with commercially available kits (COBAS 311, Roche Diagnostics GmbH, Mannheim, Germany). Low-density lipoprotein cholesterol (LDL-C) was quantified using the COBAS 311 analyzer via the particle-enhanced immunoturbidimetric method. Serum glucose concentrations were measured enzymatically using the hexokinase method (Roche Diagnostics GmbH, Mannheim, Germany). Blood HbA1c levels were assessed with the COBAS 311 analyzer using the particle-enhanced immunoturbidimetric method.

### 2.4. Measurement of Serum Endocan and Soluble Cell Adhesion Molecule (sCAM; ICAM-1, VCAM-1) Concentrations

Endocan, sICAM-1, and sVCAM-1 concentrations were quantified using a sandwich enzyme immunoassay ELISA kit (Bioassay Biotechnology Laboratory, Cat No: E3160Hu, Cat No: E0203Hu and Cat No: E0212Hu, Shanghai, China). The absorbance of the samples was quantified at 450 nm utilizing a spectrophotometer equipped with a microplate reader (VERSA max, Molecular Devices, San Jose, CA, USA). The intra-assay coefficients of variation for the endothelium parameters in these tests were 5.13%, 6.24% and 6.03%, respectively. The endocan and sVCAM-1 results were quantified in ng/L, whereas the sICAM-1 result was quantified in ng/mL.

### 2.5. Statistical Analysis

The results were analyzed utilizing IBM SPSS Statistics for Windows (version 23.0; IBM Corp., Armonk, NY, USA). The Shapiro–Wilk test was employed to evaluate the distribution of variables. Normally distributed data are represented as mean ± standard deviation, while non-normally distributed variables are represented as median (interquartile range, IQR). Student’s t-test was utilized for pairwise comparison of groups with normal distribution, whereas the Mann–Whitney U-test was employed for pairwise comparison of groups with non-normal distribution. Each MetS group was divided into three equal subgroups according to tertiles of MetS-Z score values, then evaluated using the Kruskal–Wallis test. Participants with low MetS-Z scores were assigned to Group 1, those with intermediate scores to Group 2, and those with high scores to Group 3. To investigate potential associations between MetS severity and endothelial dysfunction markers, the MetS group was stratified into tertiles based on MetS-Z score values, using median and interquartile ranges to define cut-off points. This approach facilitates subgroup comparison across increasing levels of cardiometabolic burden and is commonly used in epidemiologic studies involving continuous severity indices. Analyses were conducted using the MedCalc Statistical Software version 19.1 (Medcalc software BVBA, Oostende, Belgium), and the results were assessed via receiver operating characteristic (ROC) curve analysis. Moreover, the Spearman test was employed to assess the correlations between variables that do not conform to a normal distribution, whereas the Pearson test was utilized to analyze the correlations between variables that adhere to a normal distribution. The sample size was determined utilizing the G*Power Software 3.1 (Heinrich-Heine University, Düsseldorf, Germany). To identify a significant difference between the groups based on endocan levels with a moderate effect size (Cohen’s d = 0.8), a minimum sample size of 38 individuals was necessary for both the control and MetS groups (α = 0.05, 1 − β = 0.80). Statistical significance was evaluated at *p* < 0.05.

## 3. Results

### 3.1. Demographic Information of the Research Cohorts

The fundamental features, fasting lipid profile, and glucose and insulin levels of the study groups participating in the research are summarized in Table 1. No notable variation in gender was detected between the MetS and control groups (*p* = 0.206) (Table 1). The TG, TC, and LDL-C levels in the MetS group were considerably elevated compared to those in the control group (all *p* = 0.0001; Table 1). Marked disparities in endothelial parameters were noted between the MetS and Control groups (Table 1). The levels of endocan, sICAM-1, and sVCAM-1 in the MetS group were significantly elevated compared to the control group (*p* = 0.0001, 0.003 and 0.001, respectively), as shown in Table 2. Notably, the MetS group presented markedly elevated endocan levels in comparison to the control group (*p* = 0.0001). The MetS-Z scores exhibited a progressive elevation from the Control group to the MetS group (Table 1).

The MetS cohort was categorized into three groups according to the median MetS-Z scores. Upon analyzing endocan levels across the tertiles determined by MetS-Z score, group 3 exhibited the highest levels, which were considerably elevated compared to those in groups 1 and 2 (*p* = 0.0001) (Table 2). The levels of sICAM-1 and sVCAM-1 in group 3 were also considerably elevated when compared to groups 1 and 2 (both *p* = 0.0001) (Table 2). Upon examination of additional endothelial components, the levels of sICAM-1 and sVCAM-1 in group 3 were found to be almost doubled when compared to those in group 1, with statistical significance (both *p* = 0.0001) Overall, the levels of endothelial factors in the MetS severity groups were significantly elevated, roughly doubling in group 3 when compared to group 1 (*p* = 0.0001) (Table 2).

### 3.2. Correlations Between Ox-LDL, SCORE2 and Other Parameters

**Figure 2:**
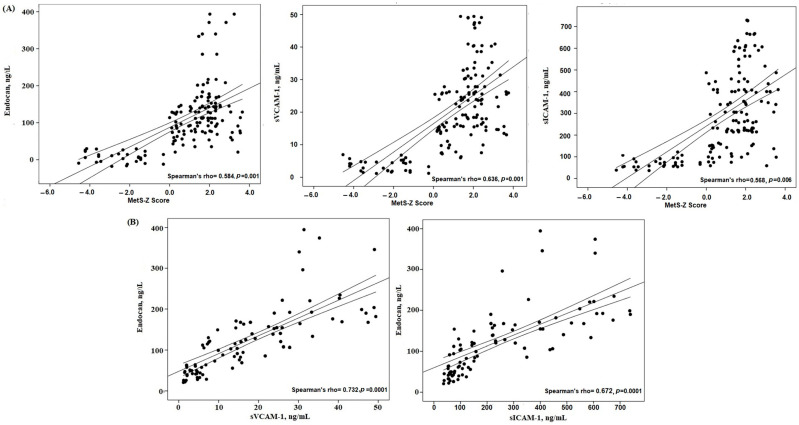
Correlations between (**A**) MetS-Z score and endothelial factors; and (**B**) endocan and endothelial factors in the study groups.

For better comprehension of the relationships between the MetS-Z score and endocan, along with other endothelial variables, Spearman’s correlation coefficients were calculated. The MetS-Z score showed substantial positive correlations with endocan and endothelial variables (Figure 2). Figure 2 illustrates the robust positive association between the MetS-Z score and endocan (r = 0.584, *p* = 0.0001). The level of endocan also exhibited positive correlations with other endothelial factors (sICAM-1, sVCAM-1) (all *p* = 0.001).

### 3.3. Receiver Operating Characteristic Analyses

**Figure 3:**
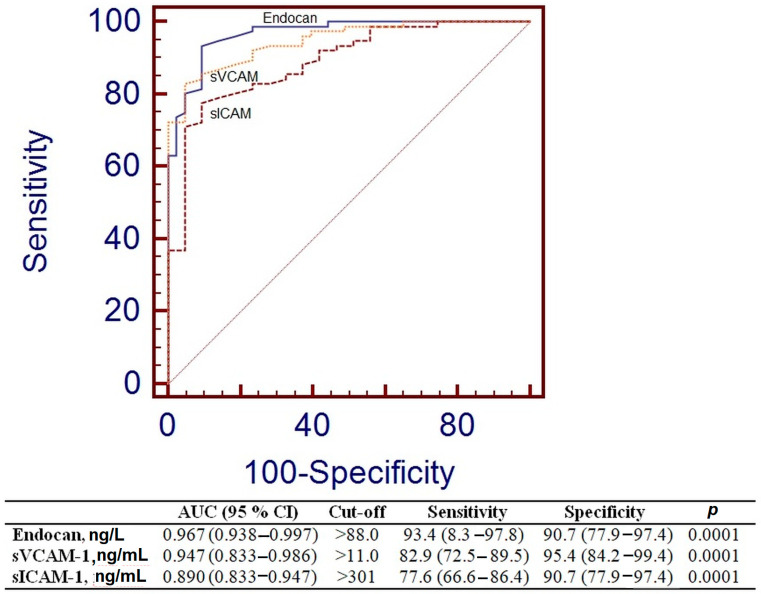
Receiver operating characteristic curve analysis for endocan, sVCAM-1, and sICAM-1.

To assess the efficacy of endocan and other endothelial variables in differentiating the control group from the MetS group, the Area Under the Curve (AUC), sensitivity, and specificity were calculated, as shown in Figure 3. Endocan exhibited markedly elevated AUC values (AUC = 0.967, *p* = 0.0001), and the threshold for endocan was more than 88.0 ng/L.

## 4. Discussion

The main results of this study regarding the MetS group and the tertile MetS Severity-Z score subgroups are as follows: (a) serum endocan levels were significantly elevated in the MetS tertile 3 subgroup compared to tertile 1 by approximately two-fold (Table 2); (b) additional endothelial factors (sICAM-1 and sVCAM-1) also exhibited significant increases and were significantly associated with endocan levels in the study groups (Table 2 and Figure 2). Recent studies have shown that endocan levels are increased in many diseases, especially in individuals with coronary artery disease, hypertension, diabetes mellitus, obesity, and chronic kidney disease [11,12,13,21]. Studies have established that serum endocan levels were markedly elevated in individuals with acute coronary syndrome relative to the control groups [22,23]. Previous studies have shown that increasing MetS severity is associated with atherosclerosis, and that blood endocan levels are significantly and consistently higher in patients with coronary artery disease [21,22,24]. On the other hand, our study is the first to investigate serum endocan levels in metabolic syndrome patients in association with their MetS-Z scores; in particular, endocan levels in MetS patients were assessed with respect to tertile values established according to the MetS severity-Z score, allowing us to more effectively assess the correlation between MetS and endothelial dysfunction within the MetS group. In the abovementioned research, the reported serum endocan levels were comparable to our findings. Moreover, a significant finding of our study is that an increase in MetS-Z score is correlated with an increase in the endocan level, with a nearly two-fold increase noted between the lower and higher tertiles in the MetS group (Table 2). These findings suggest several pathways to elucidate the role of endocan in metabolic syndrome, particularly suggesting that quantitative evaluation of MetS severity could be more effective in precisely forecasting the risk of endothelial dysfunction and cardiovascular disease. However, while this association is evident, the causal direction of the relationship between elevated endocan levels and endothelial dysfunction remains uncertain and warrants further exploration. Although our findings demonstrate a strong association between elevated serum endocan levels and MetS severity, the directionality of this relationship remains uncertain due to the cross-sectional design of the study. It is unclear whether increased endocan levels act as a driver of endothelial dysfunction or represent a downstream marker of ongoing vascular injury. Mechanistically, endocan is known to be upregulated by pro-inflammatory cytokines and oxidative stress, which are common features of metabolic syndrome. These factors suggest that elevated endocan may reflect an adaptive or compensatory endothelial response to cumulative metabolic burden. However, recent evidence also indicates that endocan itself may promote endothelial dysfunction by enhancing leukocyte adhesion, increasing vascular permeability, and modulating integrin signaling pathways [11,15]. Therefore, it is plausible that endocan plays a dual role—both as a marker and a potential mediator—of endothelial damage in MetS. Longitudinal and mechanistic studies are warranted to elucidate these causal relationships.

Endocan may aggravate vascular cell failure in metabolic syndrome patients by promoting activation and cell adhesion, and has been associated with endothelial dysfunction in many clinical scenarios [12,25]. Our data suggest that an elevated MetS-Z score is correlated with increased endothelial activity, possibly leading to the endothelial dysfunction linked to MetS (Table 2). The adherence of leukocytes through cell adhesion molecules and their corresponding ligands is promoted by inflammation mediated by cytokine signaling pathways, which have been demonstrated to promote the production of endothelial parameters such as VCAM-1 and ICAM-1 in the endothelium. Researchers have demonstrated that endocan regulates the production of adhesion molecules and integrin-mediated cell adhesion [14,26]. It has been demonstrated to enhance the production of ICAM-1 and VCAM-1, which facilitate the adherence of monocytes to endothelial cells, elevate inflammation, and augment vascular proliferation, permeability, and leukocyte migration. Conversely, endocan may impede leukocyte adherence by adhering to inflammation markers, thereby obstructing the interaction with ICAM-1 [27]. These findings demonstrate that endocan can expedite endothelial cell dysfunction through the enhancement of inflammation, cell adhesion, and oxidative damage [15,28,29]. Our results indicated that sICAM-1 and sVCAM-1 levels were significantly elevated in the MetS group relative to the control group, with a steady increase observed from subgroup 1 to subgroup 3 delineated according to the MetS-Z scores (Table 2); in particular, a nearly two-fold increase in the levels of these endothelial markers was observed between these subgroups (Table 2). The increases in the levels of endocan and endothelial parameters appeared to be gradual between the groups. Obtaining MetS scores over time facilitates the dynamic monitoring of MetS severity, allowing participants to be categorized based on MetS severity and allowing for the implementation of various interventions to avoid consequent CVD. Collectively, these findings suggest that higher endocan levels in MetS patients may be associated with increased activation of endothelial cells, resulting in increased levels of circulating adhesion molecules. Recent investigations have emphasized the complex role of endocan in facilitating endothelial dysfunction via inflammatory and oxidative pathways. Wang et al. demonstrated that endocan functions as a biomarker for endothelial activity and also influences intracellular oxidative signaling pathways in vascular endothelial cells [12]. The molecular mechanisms by which systemic metabolic stress, defined by MetS, induces endocan expression and endothelial injury are inadequately understood. Further studies using in vitro endothelial cell models exposed to serum from MetS patients would elucidate whether circulating factors directly stimulate adhesion molecule expression, enhance reactive oxygen species (ROS) production, or modify nitric oxide bioavailability. This mechanistic study could corroborate the prognostic function of endocan and enhance its therapeutic applicability as a functional biomarker in MetS-associated vascular disease.

Certain investigations have observed substantial associations between endocan levels and sICAM-1 and sVCAM-1, underscoring the roles of endocan in vascular disease and inflammation [12,30]. Additional research has validated the idea that endocan is favorably linked with ICAM-1 in MetS. Moreover, clinical studies have established a favorable correlation between endothelial dysfunction and endocan levels [12,22,31,32]. In this context, the present investigation revealed a noteworthy finding: the MetS-Z score has a substantial positive correlation with serum endocan levels, as well as with the levels of other endothelial and inflammatory factors (Table 2, Figure 2). Collectively, our findings suggest that the elevated endocan levels in the MetS group are correlated with the activation of endothelial cells, subsequently leading to increased levels of blood adhesion molecules (ICAM-1, VCAM-1). This may indicate that an elevated endocan level (along with the other endothelial variables observed in the MetS group) could be regarded as a significant risk factor for increased MetS severity. Although our study focused on endothelial dysfunction markers, it is well recognized that endothelial activation occurs in parallel with chronic low-grade inflammation in MetS. Pro-inflammatory cytokines such as TNF-α and IL-6 have been shown to upregulate endocan expression via NF-κB signaling pathways [12]. Therefore, integrating cytokine measurements into future studies could provide additional insight into the role of systemic inflammation in modulating endocan levels. Evaluating correlations between endocan and inflammatory cytokines may help determine whether endocan serves as a surrogate marker not only of endothelial stress but also of the inflammatory status in metabolic syndrome.

The observed stepwise increase in serum endocan levels across MetS-Z score tertiles may reflect a progressive endothelial activation associated with worsening metabolic derangement. Endocan, also known as endothelial cell-specific molecule-1 (ESM-1), is a dermatan sulfate proteoglycan secreted by activated endothelial cells in response to pro-inflammatory and angiogenic stimuli such as TNF-α, IL-1β, and VEGF. Functionally, endocan participates in leukocyte adhesion modulation, vascular permeability, and endothelial integrity [11,12]. Elevated endocan levels have been proposed not only as a biomarker but also as a mediator of endothelial dysfunction, via its regulation of ICAM-1/VCAM-1 expression and oxidative stress pathways. This is particularly relevant in the context of MetS, where chronic low-grade inflammation and insulin resistance are known to impair endothelial homeostasis. The nearly two-fold increase in endocan levels from the lowest to highest MetS-Z tertiles in our study may therefore represent a dynamic biological response to cumulative metabolic and inflammatory burden. These findings align with recent reports suggesting that endocan may contribute to vascular remodeling and atherogenesis in cardiometabolic disease. Future mechanistic studies, including in vitro models using patient sera, are warranted to elucidate the causal role of endocan in metabolic syndrome-associated endothelial injury.

ROC analysis was performed to evaluate the suitability of the levels of endocan and blood adhesion molecules as biomarkers of endothelial dysfunction in MetS. The endocan ratio exhibited the highest potential (AUC = 0.967, *p* = 0.0001), followed by the sVCAM level (AUC = 0.947, *p* = 0.0001) (Figure 3). The concentration of endocan demonstrated performance akin to recognized endothelial factors in the MetS group. Thus, endocan may be employed for the assessment of endothelial dysfunction in MetS patients.

The limitation of our study is the relatively small sample size, particularly within the MetS-Z tertile subgroups, may limit the statistical power for detecting subtle differences or interactions and restrict the generalizability of the findings. Although the study was adequately powered for the primary comparisons, larger studies are needed to validate our observations. Second, the cross-sectional design precludes any inference of causality between elevated endocan levels and the severity of MetS or endothelial dysfunction. Third, being a single-center study, potential institutional or demographic biases cannot be excluded. Another important consideration is that the present study was designed as a cross-sectional observational analysis, and thus cannot determine whether increased endocan levels are a cause or consequence of elevated MetS severity. The significant associations observed do not imply causality. Future studies should include longitudinal analyses to determine the temporal relationship between endocan elevation and progression of MetS.

## 5. Conclusions

This study is the first investigation demonstrating that serum endocan levels are markedly elevated and independently correlated with the MetS severity score and endothelial variables in MetS patients. The elevated endocan levels, along with increases in endothelial factors, provide evidence for endothelial activation in metabolic syndrome patients. It can be hypothesized that elevated endocan levels in the context of MetS may facilitate endothelial cell failure. Furthermore, the endocan level was determined as a potentially valuable and dependable factor for the assessment and forecasting of MetS severity. However, additional research is necessary to reliably determine which factors contributed to the obtained results.

## Figures and Tables

**Table 1:** Comparisons of the values expressed in the study groups.

Parameter	Control *n*:50	MetS *n*:120	*p*
**Gender (F/M)**	22/28	53/67	0.201
**Age (years)**	42.0 ± 6.76	45.6 ± 8.42	0.586
**BMI (kg/m^2^)**	23.8 ± 2.68	29.3 ± 4.75	0.001
**WHR**	0.782 ± 0.061	0.835 ± 0.086	0.022
**WHtR**	0.415 ± 0.037	0.528 ± 0.057	0.001
**Glucose (mg/dL)**	89 ± 2.15	112 ± 3.28	0.002
**Insulin (μIU/mL)**	8.21 [6.58–9.81]	10.5 [8.96–13.7]	0.006 *
**HOMA-IR**	1.52 [0.918–2.53]	2.38 [1.14–3.81]	0.019 *
**TG (mg/dL)**	95.0 [71–128]	188 [167–221]	0.0001 *
**TC (mg/dL)**	172 [159–188]	241 [220–269]	0.0001 *
**HDL-C (mg/dL)**	59 [48–65]	48 [37–56]	0.025 *
**LDL-C (mg/dL)**	50.0 [43.0–60.0]	53 [47–59]	0.0001 *
**SBP (mmHg)**	110 [105–120]	120 [110–130]	0.028 *
**DBP (mmHg)**	70 [70–80]	80 [70–85]	0.258 *
**MetS-Z Score**	−3.41 [−4.41–(−2.88)]	2.16 [1.88–3.64]	0.0001 *

*p* shows differences between Control and MetS according to Student’s t-test. Data are expressed as mean ± SD. * *p* shows differences between Control and MetS according to Mann–Whitney U-test. Data are expressed as median (25–75% interquartile range). BMI: body mass index, WHR: waist-to-hip ratio, WHtR: waist-to-height ratio, HOMA-IR: homeostatic model assessment for insulin resistance, TG: triglyceride, TC: total cholesterol, HDL-C: high-density lipoprotein cholesterol, LDL-C: low-density lipoprotein cholesterol, SBP: systolic blood pressure, DBP: diastolic blood pressure. *p* < 0.05.

**Table 2:** Serum levels of endothelial factors in control and MetS groups, as well as MetS-Z score tertile subgroups.

	Main Groups			Subgroups			
				MetS-Z Score Tertile			
	Control (*n*:50)	MetS (*n*:120)	*p*	1 (*n*:40) 1.18 [0.952–1.26]	2 (*n*:40) 1.75 [1.40–2.01]	3 (*n*:40) 2.21 [2.09–3.50]	*p*
***Endothelial factors***							
**Endocan, ng/L**	68.8 (58.0–75.8)	150 (86.4–176)	0.0001	98.2 (83.0–105)	125 (112–132) ^a^	168 (147–198) ^a, b^	0.0001 *
**sICAM-1, ng/mL**	157 (128–169)	327 (211–810)	0.003	188 (179–253)	262 (307–510) ^a,b^	586 (447–745) ^a, b^	0.0001 *
**sVCAM-1, ng/mL**	7.24 (5.11–9.45)	17.1 (12.1–25.9)	0.001	10.0 (9.11–12.6)	18.1 (16.1–20.0) ^a,b^	29.9 (22.3–41.5) ^a,b^	0.0001 *

*p* values according to Mann–Whitney U-test. * *p* values according to Kruskal–Wallis test, post hoc Mann–Whitney U-test. Data are expressed as median (25–75% interquartile range). a, significantly different from tertile 1; b, significantly different from tertile 2 (*p* < 0.05).

## Data Availability

The data supporting this study’s findings are available on request from the corresponding author.

## Abbreviations

The following abbreviations are used in this manuscript:

BMI	Body mass index
CVD	Cardiovascular disease
DBP	Diastolic blood pressure
Endocan	Endothelial-specific molecule 1
FBG	Fasting blood glucose
HDL-C	High-density lipoprotein cholesterol
HOMA-IR	Homeostatic model assessment for insulin resistance
LDL-C	Low-density lipoprotein cholesterol
MetS	Metabolic syndrome
MetS-Z	MetS severity Z-score
SBP	Systolic blood pressure
TC	Total cholesterol
TG	Triglyceride
WHR	Waist-to-hip ratio
WHtR	Waist-to-height ratio
